# Implementation of Digital Device–Assisted and Nurse-Led Case Management to Promote Self-Management in Adults With Noncommunicable Diseases: Protocol for a Single-Arm Intervention Study

**DOI:** 10.2196/80083

**Published:** 2026-01-22

**Authors:** Raweewan Pongpoottipatchara, Kanlayawee Anonjarn, Warithorn Prawatwong, Naruemol Singha-Dong

**Affiliations:** 1 School of Adult and Gerontological Nursing, Institute of Nursing, Suranaree University of Technology, Nakhon Ratchasima, Thailand; 2 School of Community Health Nursing, Institute of Nursing, Suranaree University of Technology, 111 University Avenue, Suranari, Mueang, Nakhon Ratchasima, 30000, Thailand, 66 44223514, 66 44223506

**Keywords:** behavioral intervention, CVD prevention, cardiovascular disease, digital health, eHealth interventions, mobile health, NCD, noncommunicable disease, nurse-led intervention, self-management, vascular health

## Abstract

**Background:**

Self-management plays a vital role in noncommunicable disease prevention and control. However, it has been challenging for patients and their caregivers to identify how much their lifestyle affects their health and what level of effort they should make to reduce cardiovascular disease (CVD) risks in everyday life. Therefore, knowing their own CVD risk and daily health-related situations will provide relevant information for self-management by those at risk. The need to help individuals understand their relevant information creates an opportunity to investigate whether and how to implement a combined digital and nurse-led self-management intervention in a real-world community setting.

**Objective:**

This study aims to evaluate the effectiveness of a combined approach combining digital device support, including a smartwatch, a mobile app, and a salt meter, with nurse-led case management, on self-management behaviors and clinical outcomes.

**Methods:**

This study uses a combination of a nurse-led self-management with a digital and mobile health innovative approach, including tailored small group face-to-face education sessions, a smartwatch, a smartphone health app, and a salt meter, to increase the self-management behaviors to reduce vascular risk through designing and testing an integrated community-based strategy targeted at adults and older adults at risk of CVD in Thailand. The study uses a single-arm pretest-posttest design to assess the intervention’s effects. The intervention will consist of the following components: (1) an interactive face-to-face education session; (2) a real-time knowing your numbers strategy using a smartwatch, a smartphone health app, and a salt meter; (3) a mindfulness-based stress management strategy using Somporn Kantaradusdi-Triamchaisri technique meditation healing exercise; and (4) a self-management diary. Quantitative data will be collected using a smartwatch, a salt meter, a food diary, and questionnaires at baseline and at the end of week 6. Clinical outcomes will be assessed at baseline, primary end point (wk 12), and secondary end point (wk 24).

**Results:**

This study, funded in January 2025, will involve 45 patients. We received ethical approval on May 31, 2024, and began recruitment for participation in May 2025. Researchers will collect, analyze, and synthesize to evaluate the study procedure. We expect to complete data collection by December 2025, with the first results submitted for publication in March 2026.

**Conclusions:**

The implementation of a combined digital device and nurse-led case management may identify the use of digital health to support self-management and improve vascular health. The findings of this study will provide insights for a large-scale randomized controlled trial and for ongoing improvements in the noncommunicable disease care system.

## Introduction

### Background

Cardiovascular diseases (CVDs) are a leading cause of noncommunicable diseases (NCDs) globally. CVDs consist of ischemic heart disease, rheumatic heart disease, hypertensive heart disease, stroke, and other cardiovascular conditions [1]. CVD caused 20.5 million deaths in 2021, as reported by the World Heart Federation. The death rates from CVDs remain steady in low- and middle-income countries, where over 80% of global CVD deaths occur [2,3]. In 2021, CVD was the leading cause of death and disability-adjusted life years (DALYs) globally, accounting for 26.8% of total deaths and 14.9% of total DALYs 2,3[4]. The American Heart Association reports a significant rise in CVDs worldwide, with heart attacks and strokes being 4 out of 5 causes of CVD deaths [5]. From 1990 to 2021, ischemic heart disease increased by 68%, stroke by 44%, hypertensive heart disease by 87%, and cardiomyopathy and myocarditis by 48% [5]. In Thailand, stroke has significantly increased every year, with an average of 8350 cases per year from 2563 to 2567 and increased to 21,012 cases or 15.28% from 2022 to 2023 and 20.77% death rate in 2024 [6]. Additionally, CVD prevalence in rheumatoid arthritis patients was higher in women with diabetes mellitus, hypertension, and dyslipidemia in Thailand [6].

The risk of CVD is associated with several common risk factors such as age, diabetes, high blood pressure, high cholesterol, a family history of vascular or heart disease, obesity, and smoking, such as an improper perception of health, a diminished sense of control, limited availability of health information, inadequate self-management skills, delayed and inefficient communication between health care providers and patients, and insufficient instrumental support [5,7,8]. Managing these risk factors through self-management intervention can help prevent or minimize the onset of vascular complications [6,9]. Therefore, encouraging self-management is crucial for preventing and controlling CVD. Self-care is a naturalistic decision-making process that addresses both the prevention and management of chronic illness, with core elements including self-care maintenance, self-care monitoring, and self-care management [10]. Self-management refers to a patient’s ability to cope with all aspects of a chronic illness, including symptoms, treatment, physical and social consequences, and lifestyle changes [6]. Several literature reviews have found that self-management strategies incorporating lifestyle modifications and behavioral interventions have been shown to improve vascular health [10-13].

Successful management of CVD risk involves interactive group education on the disease and its management, combined with regular monitoring and provision of feedback [9,10]. It also includes providing equipment and lifestyle advice supported by dietary and physical activity guidance, weight reduction, stress management, and medication adherence [11,14]. In NCD self-management, nurses play a crucial role in supporting patients with uncontrolled chronic conditions by providing health education, counseling, coaching, and support [13]. However, it has been challenging for patients and their caregivers to identify how much their lifestyle affects their health and what level of effort and intensity they should make to lower CVD risks in everyday life. Therefore, knowing their own CVD risks and daily health-related situations will provide relevant information and motivation for self-management among those at risk. Digital health interventions (DHIs) have become recognized as an effective complementary strategy [7,9,15-17] to enhance self-management support, as they overcome barriers and time constraints that traditional self-management approaches often encounter.

Interest in using smartwatches and digital technologies to develop DHIs that improve health and well-being has risen in recent years. Several reviews have reported that the effects of DHIs, including telemedicine, mobile health, and decision support tools, have enabled virtual access to clinical care, fostered positive health behaviors, and shown improvements in health outcomes and health care delivery for CVD, thereby improving CVD health [7,15,17-19].

Even though DHIs have proven to be effective in enhancing self-management behaviors and promoting clinical health outcomes, DHIs have been challenging in resource-limited settings [20]. The combination of DHIs and health care providers’ interventions can potentially help patients with NCD improve adherence to self-management when compared with stand-alone DHIs [7]. A systematic review found that smartwatch interventions in the everyday lives of patients aged ≥50 years help support lifestyle changes, improve treatment adherence, and improve clinical outcomes for CVD [19,21]. Recent smartwatch technology, which connects to a mobile app, has enabled lifestyle, goal tracking, and interactive communication interventions, thereby fostering virtual access to clinical care and promoting positive health behaviors that lead to achieving clinical outcomes [17,21,22]. The evaluation of the implementation strategy in smartwatch and digital health research studies, especially in Thailand, is limited [21]. This limitation presents an opportunity to investigate whether and how a combined DHI and nurse-led self-management intervention promotes vascular health in a real-world, resource-limited setting with diverse health determinants in Thailand.

Poor eating habits are a significant risk factor for NCD, especially CVDs. The Dietary Approaches to Stop Hypertension (DASH) diet emphasizes vegetables, whole grains, lean protein, and low-fat dairy, while limiting sodium, unhealthy fats, and sugary beverages. It is particularly beneficial for cardiovascular health, especially for those with hypertension [23]. Research shows it effectively lowers blood pressure, with reductions of −3.89 mm Hg systolic and −2.19 mmHg diastolic compared to control diets [24]. Additionally, the DASH diet has a positive impact on other cardiovascular risk factors, including blood pressure, triglycerides, cholesterol levels, body weight, and BMI [23,25,26]. The duration of DASH diet interventions varies. A study found that the DASH diet reduced 10-year atherosclerotic CVD risk scores by about 10% in just 8 weeks [27]. The DASH-sodium trial noted significant reductions in cardiac injury and inflammation markers over 12 weeks [28]. Additionally, a systematic and meta-analysis review suggested that a duration of 12 weeks or more is particularly effective for achieving reductions in body weight, BMI, total cholesterol, and low-density lipoprotein cholesterol [29].

Overall, these findings suggest that the DASH diet can offer significant cardiovascular benefits within 8 to 12 weeks, with longer adherence potentially enhancing these effects. However, this study uses a 12-week intervention to assess its outcomes. Participants in this study will use digital devices only for the first 6 weeks, then engage in self-management activities for another 6 weeks. These findings highlight the significant benefits of the DASH diet in enhancing cardiovascular health by addressing multiple risk factors and biomarkers associated with hypertension and CVD. Overall, the DASH diet stands as a well-supported dietary intervention for improving cardiovascular health in individuals with hypertension, with evidence indicating its broader applicability across various populations and health conditions.

Excessive salt intake is a significant risk factor for hypertension, a leading cause of NCDs in Thailand [30,31]. Studies suggest that each 1 g increase in sodium intake above the reference level would result in a 2.11 mmHg increment in systolic blood pressure (BP) and a 0.78 mm Hg increment in diastolic BP [31]. Salt meter, a device used to detect sodium content in daily food, is crucial in Thailand for testing and managing NCD, especially hypertension and CVD [31]. Since a salt meter can tell individuals about their sodium intake, it helps them manage their salt consumption habits. A randomized controlled study found that using a salt meter for self-monitoring of salt intake, in conjunction with dietary education, was superior to education alone in patients with hypertension, resulting in better BP control [31].

Excessive salt intake significantly increased the risk of hypertension, stroke, and total CVD [32], a leading cause of NCDs in Thailand [23,24]. A salt meter is crucial for testing and managing NCDs in Thailand, particularly hypertension and CVD [24].

As tailored self-management services and digital health devices are not equally accessible, we need to investigate if, when used with nurse-led self-management, a generic smartwatch and a salt meter can be used to supply individuals with mild to moderate CVD risk with the estimated reference information to guide daily self-management behaviors and whether the use of digital devices for a short duration can offer a carry-on estimation to guide self-management behaviors to lower CVD risk.

This study will address the gap between face-to-face and DHIs for improving self-management behaviors in urban and suburban settings in Thailand by empowering individuals with NCDs to take proactive steps to manage and engage in self-care in their daily lives. This study aims to evaluate the effectiveness of tailored, context-specific education and mobile health engagement, including the use of smartwatches, smartphones, and salt meters, in promoting self-management behaviors. Tailored, case-specific, and context-specific education, digital engagement, real-time information supply, and free primary care access are all critical for improving perceptions of risk, increasing literacy in, and motivating improvements in CVD-related health behaviors. General and reasonable smartwatches may help people get a snapshot of their daily health information, which they will use as a reference to adjust their diet, exercise, stress management, and sleep habits to meet their anticipated everyday milestones. The findings may offer practical guidance for nurses and health care professionals managing CVD in Thailand and contribute valuable insights for shaping future health care policies.

### Objectives

This study aims to investigate whether a combined approach involving digital device support, including a smartwatch, a mobile app, and a salt meter, coupled with nurse-led case management, can enhance self-management behaviors and improve clinical outcomes.

### Hypotheses

We have three primary hypotheses. First, we hypothesize that patients with chronic diseases who are at mild to moderate cardiovascular risk will report overall acceptable satisfaction with the mobile digital interventions. Second, we hypothesize that participants will use digital-based information as a reference to adjust their self-management behaviors in response to the nurse’s tailored interventions over 6 weeks. Third, we hypothesize that participants will exhibit improved self-management behaviors at weeks 6, 12, and 24, better clinical outcomes, and reduced CVD risks at both the end of week 12 (primary endpoint) and 3 months posttreatment (week 24; secondary end point).

## Methods

### Study Design

**Figure 1.**
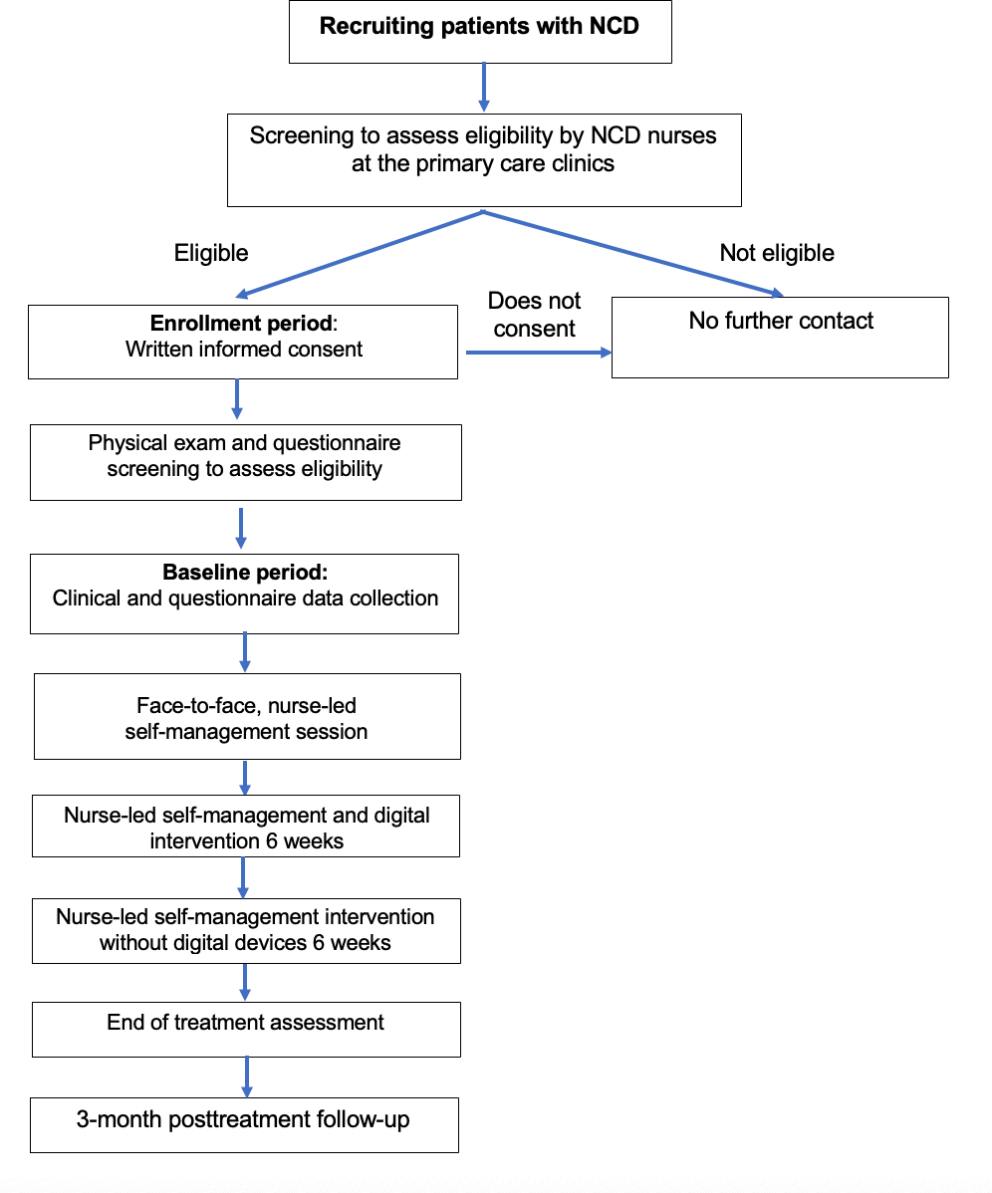
Study design flowchart. NCD: noncommunicable disease.

This study uses a single-arm pretest-posttest design to assess the effects of the intervention (Figure 1). The study was perofrmed per the SPIRIT checklist (Checklist 1)[33].

### Study Components

#### Overview

**Figure 2.**
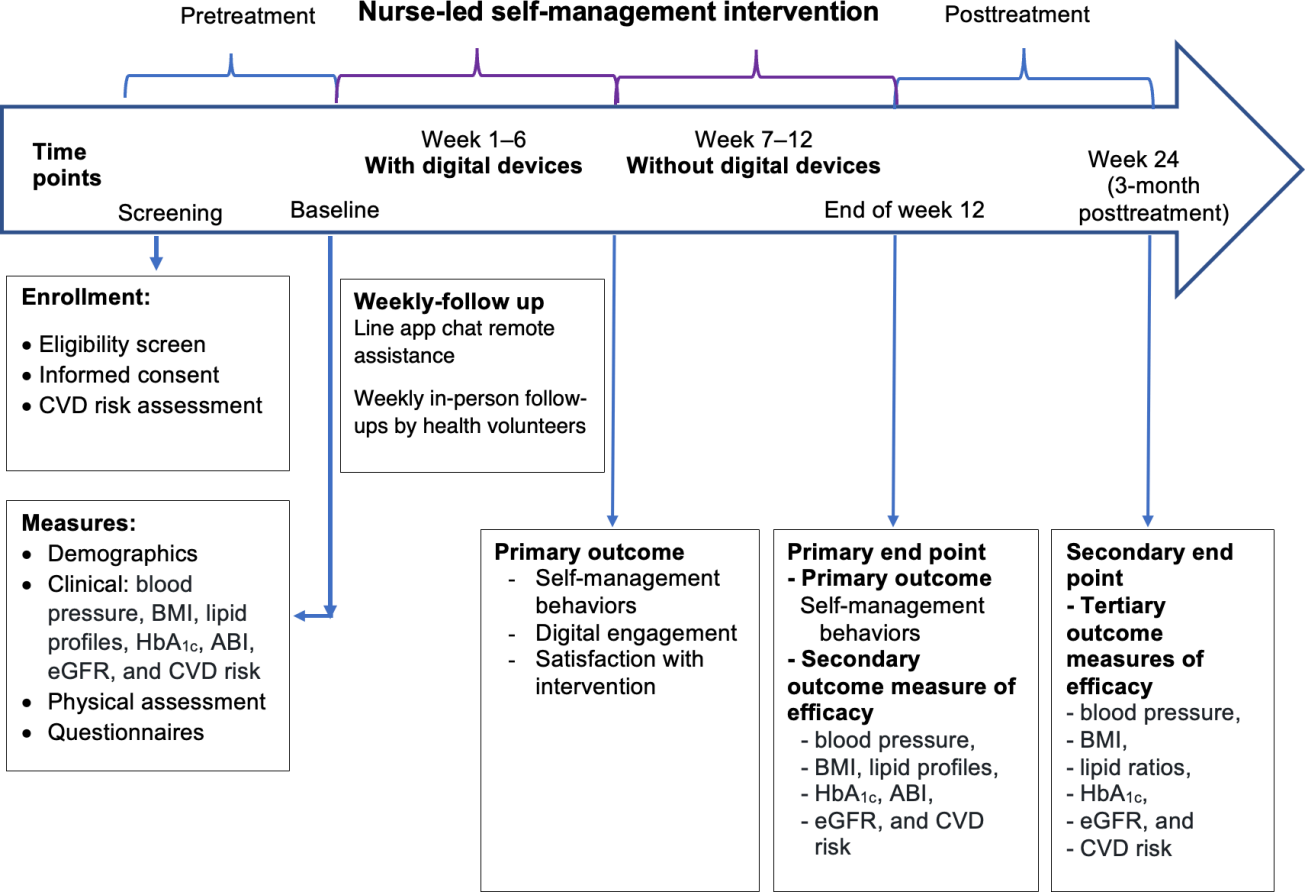
Timeline of enrollment, assessments, intervention, and follow-up. ABI: ankle-brachial index; CVD: cardiovascular disease; eGFR: estimated glomerular filtration rate; HbA_1c_: hemoglobin A_1c_.

To increase self-management behaviors and reduce vascular risk among adults and older adults at risk of CVD, this study provides interactive group education on vascular health and its management, accompanied by condition monitoring and feedback (Figure 2).

The intervention will consist of the following components: (1) a nurse-led tailored self-management session with an interactive face-to-face education, (2) a real-time knowing your numbers strategy using a smartwatch, a smartphone health app, and a salt meter, (3) a mindfulness-based stress management strategy using the Somporn Kantaradusdi-Triamchaisri (SKT) meditation healing exercise, and (4) a self-management manual. We will ask all participants to engage in self-management behaviors for 6 weeks and to continue their self-management practice without digital devices for an additional 6 weeks.

#### A Nurse-Led Tailored Self-Management Session

This management session will involve (1) a health assessment to identify the needs of participants, (2) an interactive face-to-face education and self-management consultation responsive to individual health needs, (3) a mindfulness-based SKT meditation healing exercise training, and (4) a digital device training. A family doctor will conduct a physical examination, and a nurse will conduct a structured interview. A self-management session focuses on lifestyle management based on the DASH diet, tailored to address the individual needs of individuals with diabetes and chronic renal disease. It includes physical exercise, sleep improvement, and training in self-management skills.

#### A Real-Time Knowing Your Numbers Strategy

This study component comprises the use of a smartwatch, a smartphone health app, and a salt meter. We will ask participants to use these digital devices for 6 weeks.

##### A Smartwatch: Huawei Watch Fit 3

This study will use the commercialized Huawei Watch Fit 3 and its associated HUAWEI Health app (Huawei Technologies Co, Ltd). Study participants will use this smartwatch and its smartphone app to collect daily reference data on physical activity, sleep quality, heart rate, and stress levels, which will guide their daily self-management efforts and actions in line with their target setting with the nurses. The device features a built-in interactive function that allows each participant to set goals for exercise, physical activity, and sleep. The health app helps participants track their progress and compare milestones on a weekly and monthly basis. For instance, if a participant misses a walking target, they can take additional steps in their daily routine. Participants can continue using the health app to track their physical activity after using this wearable device. Similarly, participants can use sleep information from a prior night to improve their sleep hygiene.

##### Portable Salt Measurement Device: CHEM Meter or Salt Meter

**Figure 3.**
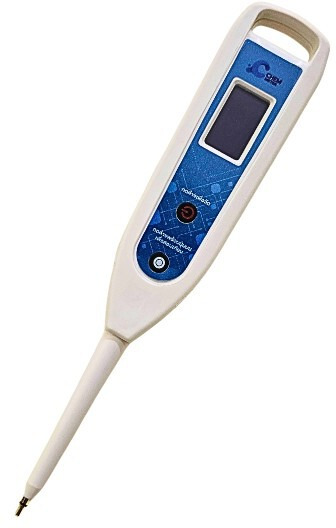
Chem meter m device used in this study (Quality Assembly Co, Ltd).

**Figure 4.**
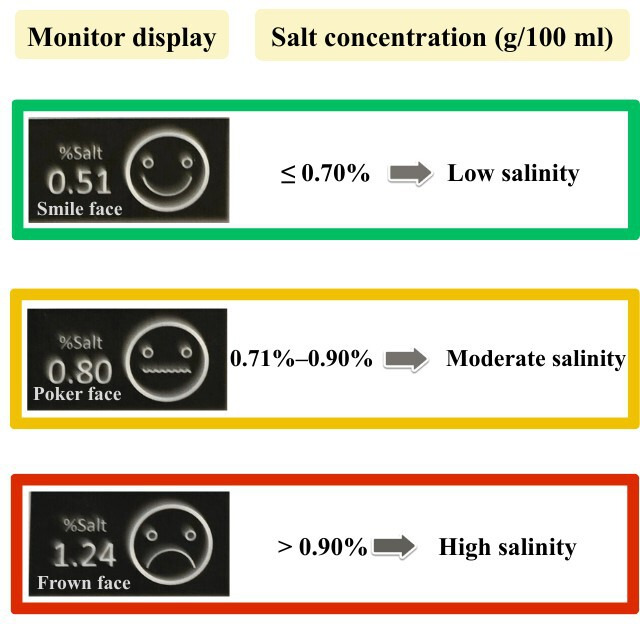
Examples of the CHEM meter facial display intended to show sodium chloride (NaCl) concentration in the interested food; smile for the NaCl <0.7%, poker face for NaCl of 0.7%‐0.9%, and frown for NaCl >0.9%.[35]

CHEM Meter (Quality Assembly Co, Ltd; Figure 3) is a portable salt measurement device using a parts per million/total dissolved solids meter with total dissolved solids expressed in parts per million by measuring the electrical conductivity of sodium chloride (%NaCl) in grams per 100 milliliters (g/100 ml) in food and converting into parts per million unit [34]. The devices use the alternating electrical current, which has a high frequency, to accurately determine the concentration of sodium chloride and display the result in emotional facial graphics; smile, poker face, and frown for sodium chloride content between <0.7%, 0.7%‐0.9%, and >0.9%, respectively [31] (Figure 4). This design is easy to understand for all users. The researchers require all participants to use a salt meter to test sodium levels in their food before meals and adjust their food consumption accordingly to limit daily sodium intake based on their individual health needs.

### The SKT Meditation Healing Exercise

The SKT is a meditation-based intervention aimed at improving vascular health, particularly in individuals with hypertension [36]. This technique involves a standing deep breathing meditation exercise, which has significantly reduced both systolic and diastolic BP in patients with uncontrolled hypertension and diabetes. The SKT technique is effective when practiced for 20 minutes, twice daily, for 8 weeks. This approach provides a nonpharmacological option for managing BP, a critical factor in maintaining vascular health. A study involving 43 patients with uncontrollable hypertension showed that those who practiced the SKT technique experienced a significant reduction in both systolic and diastolic BP after 8 weeks, compared to a control group that only received medication [37].

### A Vascular Care Self-Management Manual

The self-management manual is a color-printed A5-sized manual. The researchers developed this manual based on the study’s self-management components to assist the study participants in maintaining the desired management. The manual includes easy-to-use information on the DASH diet, a compatible list of local foods, a step-by-step pictorial SKT meditation healing exercise, information on physical activity and exercise, and an approach to improve sleep quality. The researchers developed this manual based on a literature review. A panel of 5 experts, comprising 2 family medicine doctors, 1 NCD nurse case manager, and 2 nutritionists, reviewed the manual. The researcher revised the manual based on expert suggestions and comments before production. Participants will use digital-based information as a reference to adjust their self-management behaviors in response to the tailored interventions offered by the nurses.

Participants will receive a smartwatch, a salt meter, and printed manuals to support guidance on dietary and physical activity, stress management, and sleep management. All enrolled participants will attend a face-to-face session. The digital components last for 6 weeks. After 6 weeks, we will ask all participants to continue their dietary, physical activity, stress management, and sleep management behaviors based on the information they gained from the digital components during the previous 6 weeks. All participants will record their daily diet and exercise using the printed diary.

### Recruitment

Researchers are recruiting participants for this study from primary care clinics in Nakhon Ratchasima, Thailand. The nurses at the primary care clinics will screen potential participants to determine if they meet the inclusion criteria. If the potential participants are deemed eligible and willing to participate in the study, the nurses will refer them to the researcher. Next, the researcher will approach potential participants who meet the inclusion and exclusion criteria to inform them about the study details, obtain written informed consent, and assess their digital health literacy. Those who pass all inclusion and exclusion criteria are eligible for recruitment into the study.

### Inclusion and Exclusion Criteria

The inclusion and exclusion criteria are present in Textbox 1.

Textbox 1. **Inclusion and exclusion criteria.**

**Inclusion criteria**

- Aged ≥20 years with noncommunicable disease, both man and woman.
- Determined as having a mild to moderate risk for vascular complications, including having at least 1 condition such as uncontrolled diabetes, high blood pressure, hyperlipidemia, a family history of vascular or heart disease, or smoking, or being aged over 50 years. A nurse will assess these risks using the Thai cardiovascular risk score, while a family doctor will confirm eligibility by reviewing the medical history of patients.
- Have access to a smartphone.
- Able to use digital devices and communicate in Thai.
- Having the intention to change self-management behaviors according to verbal screening.
- Able to provide informed consent.

**Exclusion criteria**

- Acute medical condition requiring hospitalization.
- Cognitive impairment or inability to participate.
- Already enrolled in a similar program.
- Recently diagnosed with strokes, hypertensive heart disease, ischemic heart disease, heart failure, acute myocardial infarction, and psychotic disorder.
- Having inadequate digital health literacy as measured by the eHealth Literacy Scale. The cut-off points of <26 indicate inadequate digital health literacy [38].

### Setting and Participant Demographics

Participants include individuals at risk of CVDs, such as uncontrolled hypertension, hyperlipidemia, and diabetes, who live in the urban and suburban communities of Nakhon Ratchasima province of Thailand. Residents of these communities have diverse educational backgrounds and socioeconomic statuses. In this setting, the younger generation typically holds an associate’s degree; the middle group is educated primarily at a lower level, often with labor-intensive work, whereas the older generation has slightly better education, with an average ranging from an associate’s degree to a bachelor’s degree.

### Sample Size Calculation

The primary outcome of this study is the change in the self-management behavior score from baseline to 12 weeks postintervention. The sample size was calculated for a paired-samples within-group design using G*Power. Based on previous studies evaluating digital and nurse-led interventions to improve self-management in individuals with chronic conditions [27], a moderate effect size (Cohen *d*=0.5) was assumed. With a significant level of 0.05, 80% power (1-β=.80), and a Cohen *d*=0.5 effect size, the required sample size to detect a change is 34. Allowing for a 30% attrition rate, the sample size was adjusted to 45 participants.

### Data Collection Procedures

Researchers will collect information on CVD risk, sleep quality, stress levels, and food consumption, as well as self-management behaviors, using physical examinations, questionnaires, and semi-structured interviews at baseline and the conclusion of the study.

1. The research team will collect CVD risk information using health reviews and questionnaires.
2. Digital data will be collected using the Huawei Watch Fit 3, which is associated with the HUAWEI Health app. These data do not include participant identification information. The researchers will access digital health data, including physical activity, sleep patterns, heart rate, and stress levels, through the HUAWEI cloud system. Data encryption will be used to protect data from unauthorized access, theft, or modification by scrambling it into an unreadable format that can only be decrypted with a special key.
3. Sodium consumption: participants will use a CHEM meter to measure the salt concentration in their food before meals and record their sodium intake in their food diary.
4. Participants will record their mindfulness activity, diet, and exercise data in a self-management diary.

Participants will be monitored weekly for 6 weeks through a health volunteer visit and a web-based survey. For the primary outcome, participants will answer questions about their self-management behaviors during (1) an initial medical screening; (2) at the end of weeks 1, 2, 3, 4, 5, and 6; (3) immediately after digital treatment at the end of week 6; and (4) week 12. For clinical outcomes, the primary end point is assessed at the end of week 12 (short-term outcomes), and the secondary end point is evaluated at week 24 (delayed outcomes) after the intervention is initiated. Clinical outcomes will include BP, BMI, lipid profiles, lipid ratio, HbA_1c_, ankle-brachial index, estimated glomerular filtration rate, and CVD risk. Acceptability will be assessed using a semistructured in-depth interview by researchers.

### Method of Measurement

The research instruments consist of (1) screening measurements, (2) experimental measurements, and (3) data measurements.

#### Screening Measurements

##### The eHealth Literacy Scale

Electronic health literacy is “*the ability to seek, find, understand, and appraise health information from electronic sources and apply the knowledge gained to addressing or solving a health problem*” [39]. Norman and Skinner [39] developed the eHealth Literacy Scale (eHEALS) measurement, which researchers will use to screen the digital health literacy of qualified participants. The eHEALS consists of 8 items rated on a Likert scale ranging from 1 (strongly disagree) to 5 (strongly agree). Possible scores range from 8 to 40, with a cut-off point of 26 or higher indicating adequate digital health literacy [38]. The Department of Health, Ministry of Public Health, subsequently translated the eHEALS into Thai. The Thai eHEALS has a test-retest reliability of 0.85 [40].

To ensure participants can safely and effectively engage with the digital intervention, participants will be required to score 26 or higher on the eHEALS to affirm high intent to use digital tools during the screening process. This threshold is applied pragmatically to maximize engagement and minimize attrition during the intervention phase, which is crucial for assessing the intervention’s initial efficacy signal.

##### The Thai Cardiovascular Risk Score

The Thai Cardiovascular Risk score was developed based on cohorts collected from the Electricity Generating Authority of Thailand study and validated using the 2nd and 3rd Electricity Generating Authority of Thailand cohorts, respectively [41]. The Thai health system has been practically using the Thai Cardiovascular Risk score as a reliable tool to forecast the risk of having a cardiovascular event in the future for Thais nationwide. The developer classified the risk as low risk (0%-10%), moderate risk (10%-20%), high risk (20%-30%), very high risk (30%-40%), and extremely high risk (40% or more).

### Experimental Measurement

The study will implement a self-management intervention comprising digital health components and a nurse-led face-to-face component among Thai participants. This study will use the Huawei Watch Fit 3 to collect digital data, including physical activity, sleep, heart rate, and stress level, and a CHEM (salt) meter to measure salt concentration in food.

### Data Measurements

#### The Demographic Data Form

Researchers developed this form to collect demographic data. The form includes a structured set of questions asking about gender, age, education, occupation, income, income adequacy, medical history, medication use, and underlying diseases.

#### The Dietary Behavior Screening (Sweet, Fatty, Salty) Questionnaire

The researchers will use the Dietary Behavior Screening (Sweet, Fatty, Salty) Questionnaire (DBSQ) to examine the dietary behavior of Thais with NCD. The Raipoong Academy, in collaboration with the Royal College of Physicians of Thailand, developed the DBSQ for public use as part of the Prevention & Health Promotion campaign. This measurement consists of 15 items: 5 items for sweet, 5 for fatty, and 5 for salty diet [42]. This measurement uses a 3-category Likert scale, from 1‐3 (1=“not at all” and 3=“always” for negative behaviors; and 3=“not at all” and 1=“always” for positive behaviors). The response choices for all items are standardized on a scale from 5 to 15, with scores of 6 to 9, 10 to 13, and 14 to 15 indicating mild-to-moderate, high, and critical dietary behavior risk, respectively. This study will examine the reliability of the DBSQ using 30 Thais with NCDs and obtain the Cronbach α coefficient before collecting the data.

#### Sleep Quality: The Thai Pittsburgh Sleep Quality Index

The Pittsburgh Sleep Quality Index (PSQI) is a 19-item self-report questionnaire that evaluates various aspects of sleep over the past month. The questionnaire assesses perceived sleep quality, sleep habits, such as habitual bedtimes and wake times, and sleep disturbances, including difficulty falling asleep, disordered breathing, nightmares, and pain [43]. A habitual bed partner is also required to rate 5 challenging questions about sleep, such as loud snoring, pauses in breathing, leg twitching, and disorientation during sleep. A scoring procedure generates scores for 7 distinct components: subjective sleep quality, sleep latency, sleep duration, habitual sleep efficiency, sleep disturbances, use of sleeping medication, and daytime dysfunction.

Component scores range from 0 (no difficulty) to 3 (severe difficulty), and their sum produces a global score ranging from 0 to 21, with higher values indicating poorer sleep quality. A PSQI score greater than 5 is considered indicative of poor sleep quality, based on its high sensitivity and specificity in identifying patients with sleep complaints [43]. Researchers developed the Thai-PSQI using a standardized method to measure major sleep disorders in Thai patients. The Thai-PSQI has a Cronbach α of 0.837 and test-retest reliability (intraclass correlation coefficient=0.89) [44]

#### The CVD Self-Management Behavior Questionnaire

The primary investigator developed the CVD Self-Management Behavior Questionnaire to examine self-management behaviors among Thais at risk of CVD. This measurement comprises 17 items: 4 related to exercise behaviors, 6 to dietary habits, 5 to treatment compliance (rational medication use), and 2 to smoking behaviors. This measurement uses a 6-category Likert scale, ranging from 0 to 5 (0=“not at all” and 5=“always”). The response options for all items are standardized, with higher scores indicating better CVD risk-prevention behavior. The researcher will test the CVD Self-Management Behavior Questionnaire for reliability with 30 Thais at risk of CVD and obtain a Cronbach α coefficient before collecting the data.

#### The Thai Stress Questionnaire (ST-5) or Srithanya Stress Test–5 Items

Srithanya Psychiatric Hospital developed this self-administered brief questionnaire to assess perceived stress levels [45]. The Stress Questionnaire-5 (ST-5) comprises items related to sleep problems, decreased concentration, irritability, boredom, and feelings of isolation. It measures subjective feelings over the past 2 to 4 weeks, using a 4-point Likert scale (0‐3) for each item. The total score is then categorized to indicate the level of stress experienced by the individual: 0 to 4 indicates mild stress, 5 to 7 indicates moderate stress, 8 to 9 indicates severe stress, and 10 to 15 indicates extremely severe stress. The ST-5 has a Cronbach of 0.85 [45].

#### An Automatic BP Measurement

A health volunteer will use the Omron HEM-9200T, along with the standard method for measuring BP, as outlined in the 2024 Thai Hypertension Society guidelines [46]. The researchers will require calibration of the automatic BP measurement using the clinical instrument testing center by the standard calibrator lab at the start of the intervention and during the study to ensure accuracy.

### Statistical Analysis

The analysis will include both the complete case and intention-to-treat analyses. The researcher will be using SPSS Statistics (IBM Corp) and Jamovi (GNU Affero General Public License and GNU General Public License) open-source statistical software to analyze quantitative data using the following statistical methods:

1. Descriptive statistics, including frequency, percentage, mean, and SD, were used to characterize the demographic data and sample characteristics.
2. Repeated measures ANOVA will analyze changes in self-management behaviors across baseline, week 6, week 12, and week 24. Additionally, clinical outcomes measured at baseline, week 12, and week 24, including those that show significant differences at one or more time points, will be evaluated [47]. This method is based on data that meet assumptions, including normality of the dependent variable, no outliers, sphericity (equal variances across time points), and a balanced number of observations.
3. In case data are missing or observations are unbalanced at each time point, linear mixed effects models will be used to evaluate changes in outcomes over time [48]. Researchers will set time as a fixed effect and individuals as random effects.
4. Post hoc tests will be used to accurately determine the specific differences between time points, using a Bonferroni correction adjustment [47].

The hypothesis will be tested in a hierarchical approach. The primary outcome of self-management behaviors will be tested first at the α=.05 level. If significant, the first key secondary outcome, blood pressure, will be tested, and so on. For the set of secondary outcomes contributing to the main effect claim, the values will be adjusted using the Holm-Bonferroni method to control the family-wise error rate at α=.05.

### Ethical Considerations

The study procedures follow the institutional and national ethical standards of the responsible committee on human experimentation and the World Medical Association Declaration of Helsinki. Furthermore, all researchers hold current Good Clinical Practice training certifications. The researchers will use various strategies, including a full-board review and ethical approval from the Human Research Ethics Committee of Suranaree University of Technology (approval 51/2566, dated May 31, 2024; renewed in June 2025). Second, all participants will be informed about the study’s objectives, procedures, risks, and benefits. Participation is voluntary, and participants can withdraw at any time. Third, all participants will be required to complete and sign consent forms before participating. The researchers will obtain written informed consent before screening for eligibility. Fourth, participants have the freedom to opt out at any time, before, during, or after participation, without any consequences from the health care system. Fifth, the researchers will maintain the confidentiality of all collected data in accordance with applicable data protection laws [49]. For instance, the researchers will not disclose patients’ names, initials, or hospital numbers, and they will guarantee participant anonymity. Each participant will receive travel compensation for every on-site data collection visit.

Data extraction will be performed via a secure, audited call. Participants will be given a unique study ID and code for anonymization to be used throughout the project, including for the smartwatch setup. The pseudonymous dataset will be transferred via Transport Layer Security 1.2 encryption to a dedicated server environment hosted on Huawei. Data encryption will be used both in transit and at rest, with access controls and regular security audits, to protect the data.

Research data will be stored on password-protected computers and in electronic databases. Informed consent forms will be stored separately from research data. Research data will be kept for 5 years after the study ends. Each study participant will receive 100 Thai Baht (US $3.22) for traveling compensation for each clinical visit.

## Results

This study received funding in January 2025. A total of 45 patients will participate in this study. The study received ethical approval on May 31, 2024. The ethical committee subsequently granted renewal following the start of participant recruitment in May 2025. The collected data will be analyzed and synthesized to evaluate the implementation of the study procedure. The results will inform the development of a 12-week self-management program for diet, sleep, and physical activity improvement, coordinated with a patient’s case management. This program will include both behavior change and clinical outcomes, along with meaningful feedback on participant progress. Study findings will inform the design and ongoing improvement of the NCD care system and will be published in peer-reviewed journals. The study will also examine the implications of using a commercial smartwatch to guide self-management in resource-limited settings. We anticipate completing data collection by December 2025 and submitting the first results for publication in March 2026.

## Discussion

### Expected Findings

This protocol describes a single-arm, pre- and postintervention design to evaluate the feasibility, acceptability, and preliminary effectiveness of a nurse-led digital support self-management intervention for adults with hypertension, dyslipidemia, and overweight in community settings. NCDs remain burdensome nationally, regionally, and globally, especially in low- and middle-income countries, where the self-management resource gap limits effective control [50,51]. Evidence shows promise that DHIs improve behaviors, behavioral risk factors, patient engagement, and cardiometabolic outcomes [7,52]. However, evidence from real-world community settings in the Southeast Asian region is limited. This study addresses this gap by integrating smartwatch-based monitoring and digital salt intake assessment with nurse-led self-management.

Even though a single-arm design cannot infer causality, it is appropriate to evaluate feasibility preliminarily. Feasibility and pilot studies often use single-arm study designs to assess recruitment, adherence to intervention components, acceptability, and data completeness before conducting controlled trials [53]. The single-arm study is recommended when an intervention is novel, complex, or requires refinement before large-scale testing [54]. In this perspective, single-arm studies provide a valuable estimate of within-person change, intervention responsiveness, and variability needed for future sample size calculation [55].

The intervention designed in this protocol incorporates several strengths. First, it leverages evidence-based components of chronic disease self-management, including self-monitoring, personalized case management, behavioral change support and feedback, delivered by nurses, an approach that has proven effectiveness in improving clinical outcomes [12,56,57]. Second, a smartwatch wearable device provides objective indicators of physical activity, heart rate, and sleep, contributing more accurate behavioral data than self-report [19,58,59]. In addition, nurse-led case management with digital support aligns with World Health Organization (WHO) recommendations for integrating digital tools within primary care to enhance person-centered and continuing care [60-63].

Several limitations must be acknowledged and addressed. In this early phase of a single-arm study, the study is vulnerable to secular trends, regression toward the mean, and the Hawthorne effect (behavioral reactivity due to monitoring) because there is no comparison group [64]. These limitations cannot be eliminated; it is crucial to interpret carefully and avoid making casual claims [53]. Moreover, a short duration may not capture the long-term physiological change required for NCD progression. This study also has limitations: a small patient population, limited to 1 geographic area, which may affect generalizability, and the use of commercial devices, whose accuracy may vary [60]. However, the single-arm study design, which is accepted by the US Food and Drug Administration and the European Medicines Agency, has the advantage of requiring a smaller sample size and a quicker timeline [64].

A significant limitation of this study is selection bias arising from the use of restrictive eHEALS and an intent threshold. This methodology likely filtered out individuals with lower digital proficiency who often experience greater health disparities. Consequently, the reported efficacy and feasibility signs may overestimate the true impact of the intervention in the general population. The results are generalizable only to populations with similar high levels of digital literacy and motivation. A future study will use a lower or zero eHEALS cut-off to assess real-world effectiveness and to analyze digital competence as a moderator of intervention success.

Despite these limitations, this study is positioned to contribute to the evidence on digital health integration in community-based NCD management. Identifying feasibility metrics, adherence patterns, preliminary effect sizes, and patient experiences will guide the refinement of the intervention and inform the design of the following randomized trial. This study also supports broader efforts to strengthen nurse-led digital chronic NCD management within primary care systems in low- to middle-income settings.

### Conclusions

This study aims to investigate whether a combined approach involving a nurse-led 12-week self-management intervention coupled with digital device support, including a smartwatch, a mobile app, and a salt meter for the first 6 weeks, can enhance self-management behaviors and improve nurse-led case management clinical outcomes. This intervention was developed using existing evidence-based materials and inputs from important stakeholders. The study will assess changes in self-management behaviors across baseline, week 6, week 12, and week 24. Primary outcomes, including self-management behaviors, urine albumin-to-urea ratio, BP, digital engagement, and satisfaction with the intervention, will be assessed at the end of week 6. Additionally, clinical outcomes, including BP, BMI, lipid profiles, HbA_1c_, ankle-brachial index, estimated glomerular filtration rate, and CVD risk, will be assessed at baseline, week 12, and week 24.

The results will inform the development of a randomized controlled study for a self-management program coordinated with digital components. This program will include both behavior changes and maintenance strategies, clinical outcome assessment, and participant engagement and progress.

## Supplementary material

10.2196/80083 Checklist 1 SPIRIT checklist [65]

## Abbreviations

BP: blood pressure
CVD: cardiovascular disease
DALY: disability-adjusted life year
DASH: Dietary Approaches to Stop Hypertension
DBSQ: Dietary Behavior Screening (Sweet, Fatty, Salty) Questionnaire
DHI: digital health intervention
eHEALS: eHealth Literacy Scale
HbA_1c_: hemoglobin A_1c_
NCD: noncommunicable disease
PSQI: Pittsburgh Sleep Quality Index
SKT: Somporn Kantaradusdi-Triamchaisri
WHO: World Health Organization
WHO: World Health Organization
